# Fetoscopic laser ablation *vs* standard management for Type‐II and Type‐III vasa previa

**DOI:** 10.1002/uog.70186

**Published:** 2026-02-05

**Authors:** S. Backley, R. H. Chmait, E. P. Bergh, N. Agarwal, A. Llanes, G. Hamadeh, E. Hernandez‐Andrade, A. Johnson, J. Espinoza, A. Salazar, S. Zhu, R. Papanna

**Affiliations:** ^1^ UTHealth Houston Fetal Institute, Division of Fetal Intervention, Department of Obstetrics, Gynecology and Reproductive Sciences McGovern Medical School at UTHealth Houston Houston TX USA; ^2^ Division of Maternal‐Fetal Medicine, Department of Obstetrics and Gynecology Keck School of Medicine, University of Southern California Los Angeles CA USA

**Keywords:** fetal intervention, fetoscopy, laser ablation, placenta, ultrasound, vasa previa

## Abstract

**Objective:**

The standard management (SM) for vasa previa (VP) includes antepartum inpatient admission at 28–32 weeks' gestation followed by Cesarean delivery at 34–37 weeks. Case reports and case series have reported on fetoscopic laser ablation (FLA) as an alternative management approach for Types‐II and ‐III VP. This study compared maternal and neonatal outcomes in patients with Type‐II or ‐III VP who underwent third‐trimester FLA with those who underwent SM.

**Methods:**

This was a cohort study of all antenatally diagnosed cases of Type‐II or ‐III VP identified by ultrasound at, or referred to, two large referral centers in the USA between September 2016 and December 2023. Patients undergoing elective FLA were prospectively followed in both centers, while patients in the SM cohort were selected retrospectively from a single center. The primary outcome was gestational age at delivery. Comparative analysis was performed between SM and FLA cohorts.

**Results:**

There were 67 singleton pregnancies complicated by Type‐II or ‐III VP, of which 35 (52.2%) underwent FLA. There were no differences in baseline demographics between the two cohorts. The median gestational age at delivery was 36.0 (interquartile range (IQR), 35.0–37.6) weeks in the FLA cohort and 34.4 (IQR, 33.4–35.0) weeks in the SM cohort (*P* < 0.001). The rate of vaginal delivery in the FLA cohort was 62.9%. Individuals who underwent FLA had a shorter maternal antepartum stay than did those with SM (median, 1 (IQR, 0–3) days *vs* 16 (IQR, 7–23) days; *P* < 0.001). The probability of preterm delivery was higher with SM than with FLA (hazard ratio, 0.24 (95% CI, 0.14–0.44)). The need for neonatal blood transfusion was lower in the FLA cohort than in the SM cohort (0% *vs* 18.8%; *P* = 0.009).

**Conclusions:**

Third‐trimester FLA offers an alternative to SM for pregnancies complicated by Type‐II or ‐III VP, as it is associated with delivery at a later gestational age and facilitates the option of vaginal delivery. Further research is needed to assess the efficacy of FLA and to provide adequate power to evaluate the potential benefits for both maternal and neonatal outcomes. © 2026 The Author(s). *Ultrasound in Obstetrics & Gynecology* published by John Wiley & Sons Ltd on behalf of International Society of Ultrasound in Obstetrics and Gynecology.

## INTRODUCTION

**Figure 1:**
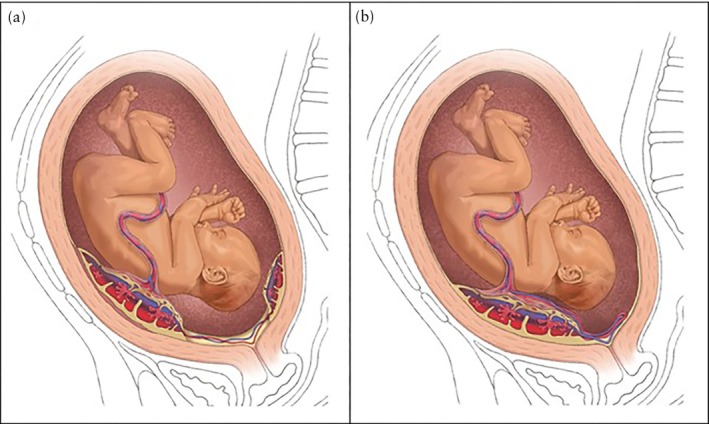
Illustration of Type‐II (a) and Type‐III (b) vasa previa.

Vasa previa (VP) is a serious pregnancy complication in which fetal vessels that are unprotected by the umbilical cord or placenta course within the fetal membranes directly over, or near, the internal cervical os. VP occurs in approximately 1 in 2500–5000 pregnancies and can cause significant perinatal mortality in cases of fetal exsanguination due to contractions or rupture of membranes^1^, ^2^, ^3^. VP is classified into three types: Type I, in which the fetal vessels course from a velamentous cord insertion to the placenta; Type II, in which the fetal vessels traverse to a succenturiate placental lobe; and Type III, in which aberrant vessels enter and exit the same placenta and travel within the fetal membranes of the lower uterine segment (Figure 1)^4^, ^5^, ^6^. There is no standardized criterion for the distance between the cervix and the fetal vessels that defines VP. Although a 2‐cm threshold has been commonly cited based on the definition of a low‐lying placenta, recent expert opinion suggests that fetal vessels located up to 5 cm from the internal cervical os are at risk during rupture of membranes or labor^7^, ^8^.

VP is detected after risk‐factor screening at the second‐trimester transvaginal ultrasound examination or during cervical‐length measurement at the anatomical ultrasound examination. The diagnosis is then confirmed in the third trimester using transvaginal ultrasound, as most cases resolve earlier in pregnancy. Improvements in prenatal diagnostic screening for VP have significantly decreased the rate of associated perinatal mortality^1^, ^3^, ^5^, ^7^, ^8^.

Although the Society for Maternal‐Fetal Medicine (SMFM) and other international societies recommend antepartum admission for a planned Cesarean delivery at 34–37 weeks' gestation, the standard management (SM) of VP varies across centers between antepartum admission and outpatient management^9^, ^10^, ^11^, ^12^. Prenatal diagnosis and third‐trimester antepartum admission significantly reduce the risk of perinatal mortality in settings in which tertiary obstetric, neonatal and anesthesia services are available^9^, ^13^, ^14^. Nonetheless, SM is limited by the potential for neonatal morbidity due to preterm delivery and uncertainty regarding optimal management^15^.

Fetoscopic laser ablation (FLA) of Types‐II and ‐III VP has been described in case reports and small series as an alternative to SM^16^, ^17^. Technically similar to FLA of placental vessels in twin‐to‐twin transfusion syndrome (TTTS)^18^, FLA confers minimal risks on the patient^19^, with preterm prelabor rupture of membranes (PPROM) being the most significant complication^20^. Prior series reported that FLA allowed outpatient management, supported the option for vaginal delivery and reduced the risk of preterm delivery^21^. To our knowledge, however, no studies have directly compared FLA with SM of VP.

This study compared Type‐II/III VP patients who underwent FLA *vs* those who received SM, to test the hypothesis that gestational age at delivery and other clinically relevant maternal and neonatal outcomes, including gestational age at antepartum admission, admission to the neonatal intensive care unit (NICU) and need for neonatal blood transfusion, differ according to the type of VP management.

## METHODS

### Study population

We analyzed data from a prospective cohort of patients treated for Type‐II or ‐III VP with FLA at two large referral centers: UTHealth Houston (UTH) Fetal Center, Houston, TX, USA, and University of Southern California (USC) Los Angeles Fetal Surgery, Los Angeles, CA, USA. Data were collected between September 2016 and December 2023 under Institutional Review Board (IRB)‐approved observational data registries at both sites (HSC‐19‐0626 and HS‐16‐00468). Patients either received a diagnosis of VP at one of the centers or were referred there for VP evaluation. Patients were eligible for FLA if they had Type‐II or ‐III VP, a singleton pregnancy, presented at 31–32 weeks' gestation (or 28–31 weeks if the patient had cervical shortening to 20–25 mm with hospitalization indicated) and had a fetal vessel within 3 cm (at the UTH center) or 2.5 cm (at the USC center) of the internal cervical os, consistent with recent reports^7^, ^22^. Eligible patients also required subjective ultrasound assessment by the surgeon to confirm that the unprotected fetal vessel(s) supplied < 20% of the placenta, which was determined by tracing the fetal vessel(s) to the placental territory and estimating that it supplied fewer than three cotyledons, as described previously^21^, ^22^. It should be noted that FLA was not available at UTH until 2020, therefore patient eligibility for the procedure, including estimation of the percentage of the placenta supplied by the fetal vessel(s), was not assessed at the UTH center until then. Exclusion criteria included Type‐I VP, fetal growth restriction, PPROM prior to FLA, multiple gestation and major fetal anomaly. Data on a subset of FLA cases reported in this study have been published previously^21^, ^22^.

To compare outcomes of FLA with SM, we analyzed data from patients who received SM for Type‐II or ‐III VP at the UTH center between September 2016 and December 2023 under an IRB‐approved registry (HSC‐19‐0626). There were no included SM patients from the USC center.

### Study procedures

All patients underwent a comprehensive ultrasound examination that included transabdominal evaluation of detailed fetal anatomy, the placenta and the umbilical cord; transvaginal measurement of cervical length; and assessment of the entire lower uterine segment and cervical os using color and pulsed‐wave Doppler analysis of any unprotected fetal vessel(s) encountered. If one or more unprotected fetal vessels crossed within 2.5 cm (at USC) or 3 cm (at UTH) of the internal cervical os^7^, ^22^, ^23^, VP was diagnosed and classified as Type I, II or III. The placental cord insertions were assessed as central (umbilical cord attached to the center of the placenta), marginal (umbilical cord attached at the edge of the placenta), eccentric (umbilical cord inserting into the placenta in a lateral site) or velamentous (umbilical cord attached to the fetal membranes instead of the placenta). The number and type of fetal vessels were recorded, and the vessel type was confirmed by color and pulsed‐wave Doppler. Ultrasound examinations were performed by certified sonographers experienced in maternal–fetal medicine and supervised by maternal–fetal medicine specialists. GE Voluson E8, E10 and E22 (GE Healthcare, Zipf, Austria) ultrasound systems were utilized.

Patients were counseled extensively about the potential benefits and risks of FLA, including: potential abandonment of the procedure if the fetal vessel(s) could not be identified or if it supplied > 20% of the placenta; possible emergency Cesarean section in case of fetal intolerance or failure to ablate the fetal vessel(s); fetal bradycardia (fetal heart rate < 110 bpm); and PPROM prior to ablating the fetal vessel(s). Patients who were eligible for and consented to FLA underwent a diagnostic endoscopy to determine the feasibility of the procedure. Cases in which FLA was deemed not possible instead received SM. These SM patients were counseled about the antenatal admission plan and the risks of Cesarean delivery and NICU admission.

### Fetoscopic laser ablation

Patients who consented to FLA received betamethasone within 1 week before the procedure to promote fetal lung maturation. Fetal weight was assessed during an additional preoperative ultrasound examination, and transvaginal ultrasound was performed to assess the cervix and fetal vessel diameter, mapping the direction and distance of the vessels from the internal cervical os.

FLA was performed with the operating room set up for emergency Cesarean delivery if necessary owing to complications. During FLA, transabdominal ultrasound was used to assess the fetal heart rate and Doppler waveform, and transvaginal ultrasound was used to map the vessel(s) and confirm cessation of blood flow. The procedure was performed under conscious sedation or regional anesthesia, depending on surgeon and patient preference. Entry into the amniotic cavity with a trocar was achieved via the Seldinger technique or direct trocar insertion, and diagnostic fetoscopy was performed using a 30° or 70° Karl Storz fetoscope (Karl Storz SE & Co. KG, Tuttlingen, Germany). If needed, amnioinfusion was performed at the discretion of the operating surgeon to facilitate maneuverability. The fetal head or breech was manually lifted out of the lower uterine segment using transabdominal pressure to better visualize the placenta and the territory supported by the fetal vessel. A Karl Storz (0° or 30°) or Wolf (Richard Wolf Medical Instruments Corporation, Vernon Hills, IL, USA) (0°) operative fetoscope was used to identify the fetal vessel(s) endoscopically while mapping the placental territory supplied by the extraplacental vessels. Subsequently, a 600‐micron non‐contact laser fiber (Surgical Laser Technologies, Montgomeryville, PA, USA) was passed through the trocar. The diode laser, with a starting energy of 20–23 W and increasing to 32–40 W, was used to target the fetal vessel(s) towards the cotyledon side. The proximal (upstream) and, if possible, distal (downstream) segments of the vessel(s) and their branches were ablated. Vessels were monitored intraoperatively using color Doppler transvaginal ultrasound to confirm successful ablation. Amnioreduction was subsequently performed to normalize the amniotic fluid volume if amnioinfusion had been performed.

Following the procedure, the patient was admitted overnight for fetal monitoring, and ablation of the fetal vessel(s) was re‐examined and confirmed via transvaginal ultrasound on postoperative day 1. If confirmed, the patient was discharged with outpatient follow‐up, as bleeding or PPROM would not be of fetal origin and would not risk fetal exsanguination. The patient was instructed to return to the center for follow‐up 1 week later for final confirmation of ablation, before returning to the care of her obstetrician, with serial growth measurement recommended. The patient was informed that she was a candidate for vaginal delivery at term following confirmation of the ablated vessel(s).

### Standard management

The SM group included only patients who underwent SM at the UTH center. Patients were admitted for hospitalization between 30 and 32 weeks, and betamethasone was administered to promote fetal lung maturation. Cesarean delivery was performed at 34 weeks, or sooner if clinically indicated, at the discretion of the managing physician. Medical records and ultrasound images were reviewed by R.P., S.B. and N.A.

### Outcomes

The primary outcome was gestational age at delivery. Secondary outcomes included admission to the antepartum unit, gestational age at antepartum admission, duration of antepartum stay, vaginal delivery rate, PPROM < 34 and < 37 weeks, NICU admission, length of NICU stay, respiratory distress syndrome, necrotizing enterocolitis, intraventricular hemorrhage and need for neonatal blood transfusion.

### Statistical analysis

Prospectively collected data were extracted from medical records and stored in the electronic research database of each institution. Continuous variables were assessed for normality using the Shapiro–Wilk test, with a threshold of *P* < 0.05. Variables that met the normality criterion are summarized as mean ± SD and were compared using Welch's two‐sample *t*‐test. Non‐normally distributed variables are summarized as median (interquartile range (IQR)) and were compared using the Wilcoxon rank‐sum test. Ordinal variables (such as gravidity and parity) are reported as median (range) and were also compared using the Wilcoxon rank‐sum test. Categorical variables were analyzed using the Pearson's chi‐square test or Fisher's exact test, based on expected cell counts.

Gestational latency was defined as the interval from the first day of the last menstrual period (LMP) to delivery. Kaplan–Meier curves, stratified by treatment arm, were generated, with time zero defined as LMP and the *x*‐axis labeled ‘gestational age (weeks)’. To focus on the period during which delivery actually occurred, curves are displayed from 25 to 40 weeks, and group differences were assessed using the log‐rank test. Cox proportional hazards regression, adjusted for cervical length, was then used to estimate the effect of treatment type on gestational latency. To control the family‐wise Type‐I error from multiple testing, we prespecified a primary family of eight clinically relevant outcomes (gestational age at delivery, gestational age at antepartum admission, duration of antepartum stay, NICU admission, NICU length of stay, neonatal blood transfusion, cervical length and history of preterm birth), selected *a priori* based on clinical relevance and prior literature. A Bonferroni correction was applied to this family of outcomes only, using a significance threshold of α = 0.00625 (0.05/8), thereby controlling the family‐wise error rate at 0.05. Data were analyzed and visualized using R version 4.3.2 (R Foundation for Statistical Computing, Vienna, Austria)^24^.

## RESULTS

### Study population

**Figure 2:**
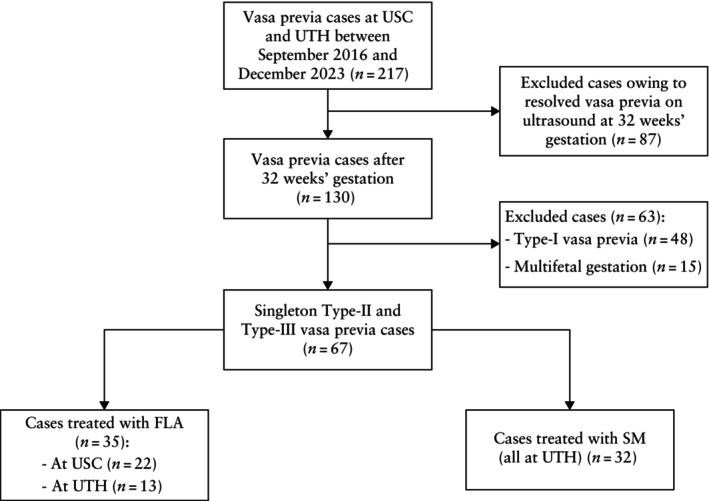
Flowchart showing selection of study population of Type‐II or ‐III vasa previa cases undergoing fetoscopic laser ablation (FLA) or receiving standard management (SM) at University of Southern California (USC) and UTHealth Houston (UTH) centers.

During the study period, 67 singleton pregnancies complicated by Type‐II or ‐III VP met the inclusion criteria (Figure 2), of which 35/67 (52.2%) underwent FLA at one of the two study centers (13/35 at UTH and 22/35 at USC). Thirty‐two patients received SM at the UTH center, two of whom were offered but declined FLA. FLA completely ablated the fetal vessel(s) on the first attempt in 33/35 (94.3%) cases. One case (2.9%) required a second procedure the following day to complete the ablation and in another case, the procedure was abandoned and an immediate Cesarean delivery was performed (2.9%)^22^.

### Characteristics by management type

Baseline demographics, risk factors and ultrasound variables were mostly similar between the two cohorts (Table 1). Median cervical length prior to admission was longer in the FLA group than in the SM cohort (*P* = 0.03); however, this difference was not statistically significant within the adjusted threshold. There was no difference between cohorts in the incidence of cervical length < 20 mm, < 25 mm or < 30 mm. Risk factors for VP, including history of preterm birth, Cesarean delivery, *in‐vitro* fertilization or placenta previa, did not differ significantly between the groups. Furthermore, VP type, fetal vessel type (artery, vein or both), number of fetal vessels and fetal vessel distance from the internal cervical os did not differ between cohorts. Type‐II VP was more common than Type‐III in each of the cohorts, accounting for 23/35 (65.7%) and 22/32 (68.8%) cases in the FLA and SM cohorts, respectively.

**Table 1:** Comparison of baseline demographics and ultrasound characteristics between patients undergoing fetoscopic laser ablation (FLA) *vs* receiving standard management (SM) for vasa previa

Characteristic	FLA (*n* = 35)	SM (*n* = 32)	*P* *
Maternal age (years)	35 ± 4	34 ± 5	0.32
Maternal BMI (kg/m^2^)	30.0 (26.0–34.0)	28.0 (26.0–31.5)	0.47
Gravidity	2 [1–17]	2 [1–5]	0.71
Parity	0 [0–7]	1 [0–4]	0.15
Race			0.20
White	21 (60.0)	15 (46.9)	
African‐American	3 (8.6)	7 (21.9)	
Asian	2 (5.7)	5 (15.6)	
Other	9 (25.7)	5 (15.6)	
History of preterm birth	5 (14.3)	3 (9.4)	0.71
History of Cesarean delivery	4 (11.4)	4 (12.5)	> 0.99
History of IVF	5 (14.3)	4 (12.5)	> 0.99
History of placenta previa	10 (28.6)	15 (46.9)	0.12
Placental cord insertion			0.02
Central	16 (45.7)	15 (46.9)	
Marginal	9 (25.7)	6 (18.8)	
Velamentous	4 (11.4)	11 (34.4)	
Eccentric	6 (17.1)	0 (0)	
Cervical length (mm)	40.5 ± 8.0	36.0 ± 8.9	0.03
Vasa previa type			> 0.99
Type II	23 (65.7)	22 (68.8)	
Type III	12 (34.3)	10 (31.3)	
Fetal vessel type			0.41
Artery only	8 (22.9)	12 (37.5)	
Vein only	12 (34.3)	8 (25.0)	
Both artery and vein	15 (42.9)	12 (37.5)	
Number of fetal vessels	2 (1–4)	2 (1–3)	> 0.99
Fetal vessel distance from internal cervical os (cm)	0.9 (0.0–1.5)	1 (0.4–1.8)	0.43

Data are given as mean ± SD, median (interquartile range), median [range] or *n* (%).

* Calculated using Pearson's chi‐square test, Wilcoxon rank‐sum test, Fisher's exact test or Welch's two‐sample *t*‐test. BMI, body mass index; IVF, *in‐vitro* fertilization.

### Primary outcome

Gestational age at delivery differed significantly between treatment cohorts. The median gestational age at delivery was 36.0 (IQR, 35.0–37.6) weeks in the FLA cohort and 34.4 (IQR, 33.4–35.0) weeks in the SM cohort (*P* < 0.001).

### Maternal outcomes by management type

Maternal antepartum and intrapartum management differed between the FLA and SM cohorts (Table 2). Admission to the antepartum unit occurred less frequently in the FLA cohort compared with the SM cohort (*P* < 0.001), and occurred later in the FLA cohort (*P* < 0.001). Of those admitted, the FLA cohort had a significantly shorter duration of antepartum stay than the SM cohort (median, 1 (IQR, 0–3) days *vs* 16 (IQR, 7–23) days; *P* < 0.001). PPROM < 37 weeks occurred in 37.1% of the FLA cohort but in 0% of the SM cohort, and PPROM < 34 weeks occurred in 14.3% of the FLA cohort but in 0% of the SM cohort. The median gestational age at delivery for the patients who had PPROM < 37 weeks was 35.1 weeks. Within the FLA cohort, 22/35 (62.9%) patients delivered vaginally; no patients who underwent SM delivered vaginally.

**Table 2:** Comparison of maternal antepartum and intrapartum management between patients undergoing fetoscopic laser ablation (FLA) *vs* receiving standard management (SM) for vasa previa

Characteristic	FLA (*n* = 35)	SM (*n* = 32)	*P* *
Admission to antepartum unit	8 (22.9)	28 (87.5)	< 0.001
Gestational age at antepartum admission (weeks)	36.2 ± 2.0	31.6 ± 2.0	< 0.001
Duration of antepartum stay (days)	1 (0–3)	16 (7–23)	< 0.001
PPROM < 37 weeks	13 (37.1)	0 (0)	< 0.001
PPROM < 34 weeks	5 (14.3)	0 (0)	0.05
Gestational age at delivery (weeks)	36.0 (35.0–37.6)	34.4 (33.4–35.0)	< 0.001
Vaginal delivery	22 (62.9)	0 (0)	< 0.001
EBL at delivery (mL)	400.0 (300.0–802.0)	757.5 (600.0–900.0)	< 0.001

Data are given as *n* (%), mean ± SD or median (interquartile range).

* Calculated using Welch's two‐sample *t*‐test, Pearson's chi‐square test, Fisher's exact test or Wilcoxon rank‐sum test. EBL, estimated blood loss; PPROM, preterm prelabor rupture of membranes.

### Neonatal outcomes by management type

Neonatal outcomes differed between cohorts (Table 3). The NICU admission rate was significantly lower in the FLA cohort than in the SM cohort (34.3% *vs* 96.9%; *P* < 0.001), and the length of stay in the NICU was significantly shorter for the FLA cohort (*P* < 0.001). Neonatal morbidity outcomes were less frequent in the FLA than in the SM cohort. No FLA cases required neonatal blood transfusion, but 6 (18.8%) SM cases did (*P* = 0.009); however, this difference was not statistically significant within the adjusted threshold. There were no fetal or neonatal deaths in either cohort.

**Table 3:** Comparison of neonatal outcomes between patients undergoing fetoscopic laser ablation (FLA) *vs* receiving standard management (SM) for vasa previa

Characteristic	FLA (*n* = 35)	SM (*n* = 32)	*P* *
NICU admission	12 (34.3)	31 (96.9)	< 0.001
NICU length of stay (days)	0 (0–6)	11 (6–22)	< 0.001
Neonatal blood transfusion	0 (0)	6 (18.8)	0.009
Respiratory distress syndrome	5 (14.3)	16 (50.0)	0.002
Necrotizing enterocolitis	0 (0)	0 (0)	—
Intraventricular hemorrhage	0 (0)	2 (6.3)	—
Birth weight (g)	2666.9 ± 510.7	2305.0 ± 518.8	0.005
Neonatal death	0 (0)	0 (0)	**—**

Data are given as *n* (%), median (interquartile range) or mean ± SD.

* Calculated using Wilcoxon rank‐sum test, Fisher's exact test or Pearson's chi‐square test. NICU, neonatal intensive care unit.

### Outcomes specific to fetoscopic laser ablation

Table 4 highlights FLA procedure‐related details and outcomes unique to the FLA cohort. Most patients underwent FLA at 31–32 weeks (31/35 (88.6%)). The median vessel diameter was 0.3 (range, 0.1–0.5) cm. Amnioinfusion was performed in 71.4% (25/35) of patients during FLA. There were no cases of fetal bradycardia during the procedure or of membrane separation during postoperative assessment. The FLA procedure was abandoned in one case, and one case required repeat FLA. Cesarean delivery was performed in 13 (37.1%) patients; indications for Cesarean delivery are shown in Table 4.

**Table 4:** Technical aspects of fetoscopic procedure and outcomes unique to fetoscopic laser ablation (FLA) cohort (*n* = 35)

Characteristic	Value
Gestational age at FLA	
28–30 weeks	2 (5.7)
31–32 weeks	31 (88.6)
> 33 weeks	2 (5.7)
Vessel diameter (cm)	0.3 (0.1–0.5)
Amnioinfusion during FLA	25 (71.4)
Operative time (min)	60 (34–125)
Membrane separation	0 (0)
Fetal bradycardia during FLA	0 (0)
Perinatal death	0 (0)
Interval from FLA to delivery (days)	33 (0–57)
Indication for Cesarean delivery	
Repeat Cesarean delivery	2 (5.7)
Elective Cesarean delivery	1 (2.9)
Low‐lying placenta	1 (2.9)
Malpresentation	1 (2.9)
Bleeding/abruption	3 (8.6)
Failed induction	4 (11.4)
Abandoned FLA procedure	1 (2.9)

Data are given as *n* (%) or median (range).

### Risk of preterm delivery

**Figure 3:**
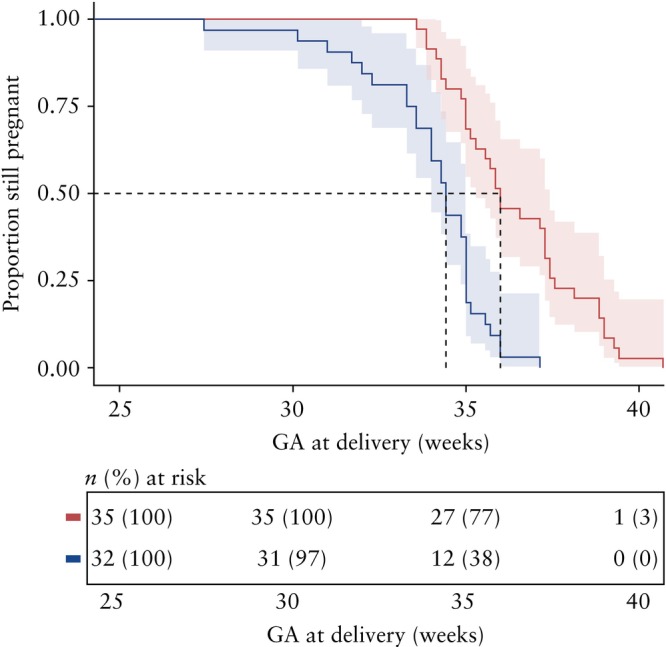
Kaplan–Meier survival curves comparing gestational latency, in weeks, between individuals who underwent fetoscopic laser ablation (FLA) (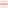) and those who received standard management (SM) (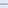), with no censoring. Dashed line represents median gestational age (GA) at delivery for each group. Significant difference was observed between cohorts (*P* < 0.0001).

Survival analysis performed using a log‐rank test found a significant difference in hazard ratios between the two cohorts (*P* < 0.0001) (Figure 3). The Cox proportional hazards model suggested a lower hazard for preterm delivery among individuals who underwent FLA compared with those who received SM (hazard ratio, 0.24 (95% CI, 0.14–0.44)). Controlling for cervical length, patients who underwent FLA had a 76% lower risk of preterm delivery compared with SM patients.

## DISCUSSION

In carefully selected patients, third‐trimester FLA for Type‐II and ‐III VP appears to be safe, with reassuring short‐term outcomes for both the mother and neonate, though numbers in this study were small and it was therefore underpowered to assess many of the neonatal outcomes. FLA was associated with lower rates of antenatal hospitalization, preterm birth, Cesarean delivery and, consequently, estimated blood loss at delivery compared with SM.

In 2007, Quintero *et al*.^16^ described the first use of FLA for the management of Type‐II VP, performed at 22 weeks' gestation. In this case, Cesarean delivery was performed at 27 weeks following PPROM. In 2014, Johnston *et al*.^25^ reported a case in which FLA was performed at 32 weeks and resulted in vaginal delivery at term. The procedure was delayed until 32 weeks in an effort to mitigate potential associated risks. By 2019, five cases of FLA for Type‐II VP management had been reported^17^, ^25^, ^26^, and larger series reporting its feasibility in Types‐II and ‐III VP followed soon afterwards^21^, ^22^, ^27^. This study, which includes some previously reported cases, is, to our knowledge, the largest series of FLA for Types‐II and ‐III VP to date and is the first to compare FLA and SM for VP.

The current SM of VP, which involves hospitalized surveillance and late preterm Cesarean delivery, aims to prevent fetal demise due to VP disruption and fetal exsanguination secondary to PPROM or preterm contractions^28^. However, though the SMFM VP consult series proposes antepartum admission at 30–34 weeks, there is currently no consensus about what constitutes optimal SM for VP^4^, ^9^, ^15^. Inpatient *vs* outpatient surveillance, timing of admission and timing of delivery vary widely between centers^28^, ^29^, ^30^. In hospitalized patients, contractions or vaginal bleeding often cause concerns that may prompt physicians to perform unscheduled Cesarean deliveries. Among SM cases in this study, 28.8% underwent an unscheduled Cesarean delivery, similar to the 37.7% rate reported by Westcott *et al*.^28^. While data recommending early antenatal admission are scarce, at least 1 in 3 patients with VP undergo an unscheduled delivery. These unexpected changes in care plans may exacerbate stress and anxiety for patients and their families^31^.

Maternal stress associated with a diagnosis of VP and potential vessel rupture remains understudied. Javid *et al*.^31^ reported on pregnant women in Australia who were diagnosed with VP and managed with SM. These women expressed constant stress and worry about the uncertainty of the perinatal outcome and fear of losing their baby. They also highlighted stress related to the inconsistent information they received about their diagnosis and management plan, reporting inconsistency between hospitals as well as between clinicians in the same hospital, particularly around the timing of delivery^31^. It is possible that, since FLA allows a definitive intervention, stress may be reduced by its use. More research is needed around maternal anxiety and stress associated with a diagnosis of VP, to establish a consensus on optimal management with consistent information^32^.

Data supporting a standardized gestational age at delivery are also lacking. Gestational age at delivery for VP ranges from 33 to 36 weeks^4^, ^15^, which is consistent with the findings of this study (~34–35 weeks). Robinson and Grobman^33^ studied the optimal timing of delivery in cases of VP via decision‐tree analysis and found that delivery at 34–35 weeks balanced the risks of perinatal mortality and preterm birth; expectant management showed no benefit beyond 37 weeks. Both of our centers and other centers across the USA use the SMFM and American College of Obstetricians and Gynecologists recommendation of delivery at 34–37 weeks^9^, ^11^, with some centers delivering closer to 34 weeks. Importantly, FLA may provide the opportunity to deliver at a later gestational age than might SM, as our survival analysis demonstrated a 76% lower risk for preterm delivery in cases of FLA, controlled for cervical length.

Research on early antenatal admission as standard practice is similarly lacking. Patients may benefit from hospitalization should an unplanned delivery be indicated, but the social cost of prolonged admission may counterbalance these considerations^34^.

Neonatal blood transfusion secondary to vessel rupture during Cesarean delivery is necessary in 3–6% of VP cases^35^, ^36^, ^37^. Previous reports include Types‐I–III VP, which may lower the actual transfusion incidence. In our series, six (19%) SM cases (four Type‐III VP and two Type‐II VP) required a neonatal blood transfusion, compared with no FLA cases. This potential benefit needs prospective validation.

The optimal timing of the FLA procedure is controversial. Thirty‐one of our cases underwent FLA between 31 and 32 weeks, after steroid administration. Intervention at such a gestational age poses a lower risk of prematurity for the neonate in case an emergency Cesarean delivery is necessary. Invasive procedures in the second trimester increase the risk of PPROM and preterm birth; thus, we recommend avoiding FLA for VP before 28 weeks to mitigate the risks reported in case reports of FLA for VP and in larger cohort studies of FLA for TTTS^16^, ^38^. Furthermore, VP may resolve by the early third trimester^39^, ^40^. Ibirogba *et al*.^41^ proposed FLA at 34–36 weeks as an alternative to an elective Cesarean section, but there is no evidence to support feasibility beyond 33 weeks. Our experience of attempting FLA at 34 weeks revealed the vessel caliber to be too large, meaning the case was abandoned and a Cesarean delivery was performed^22^. Beyond 34 weeks, the vessel diameter increases, and the increased vernix yields turbid amniotic fluid that limits visualization and, thus, procedural safety and adequate confirmation of the proximal and distal ablation of the vessel. We performed FLA in two cases at 28 weeks owing to cervical shortening (20 mm). FLA can be reasonably performed before 31 weeks in patients with cervical shortening or vaginal bleeding from other causes.

Given the steep learning curve of fetoscopic surgery, this procedure should only be attempted in highly experienced fetal therapy centers. Such centers typically have cumulative experience of hundreds of cases of FLA for TTTS, providing the technical expertise and team coordination necessary to translate this skill set to FLA for VP. Operators with a high procedural volume of TTTS‐FLA are uniquely positioned to adapt the principles of selective vessel identification, precise coagulation and management of complications to this novel application for VP.

When offering FLA for VP, clinicians should inform patients that the procedure remains innovative and investigational, using a grounded counseling approach that takes into account the ethical principles of beneficence, non‐maleficence, autonomy and justice for both the mother and fetus^42^. Based on the preliminary data of our study, this intervention appears safe, with potential maternal and fetal benefits, when performed in experienced fetal therapy centers. The ethically acceptable use of FLA for VP must include consent and rigorous oversight; otherwise, SM with planned Cesarean delivery remains the ethically preferred pathway.

### Strengths and limitations

The primary limitation of this study is that FLA was not available before 2020 at the UTH center, and the SM group did not include patients from the USC center. This opens the study to selection bias, as patients evaluated before and after 2020 who were treated by SM may not have undergone the same assessment. Furthermore, the small sample size of the study prevented analysis of all relevant maternal and neonatal outcomes. Management practices, such as ultrasound screening, education on diagnosing and evaluating VP, and implementation of routine transvaginal ultrasound, were also modified over the study period. Finally, all cases were managed as having VP. This diagnosis was confirmed during fetoscopy in the FLA group; however, in the SM group, there was no confirmation of diagnosis at delivery or on placental evaluation, so some cases in this group may not have had VP. However, evaluating the accuracy of screening for VP lies beyond the scope of this study.

### Conclusions

Although early recognition and antenatal diagnosis of VP remain the most important factors in improving outcomes, the benefits of FLA as an alternative to SM merit further investigation in select pregnancies complicated by Type‐II or ‐III VP. Future studies may begin with observational registries, followed by a randomized controlled trial to rigorously evaluate the technique with sufficient power to analyze neonatal outcomes with strict predefined criteria and postprocedural surveillance.

## Data Availability

The data that support the findings of this study are available on request from the corresponding author. The data are not publicly available due to privacy or ethical restrictions.
